# Isolation and Characterization of Extracellular Vesicles Through Orthogonal Approaches for the Development of Intraocular EV Therapy

**DOI:** 10.1167/iovs.65.3.6

**Published:** 2024-03-11

**Authors:** Justin Leung, Dimitrios Pollalis, Gopa K. G. Nair, Jeffrey K. Bailey, Britney O. Pennington, Amir I. Khan, Kaitlin R. Kelly, Ashley K. Yeh, Kartik S. Sundaram, Dennis O. Clegg, Chen-Ching Peng, Liya Xu, Sun Young Lee

**Affiliations:** 1 USC Dornsife College of Letters, Arts and Sciences, University of Southern California, Los Angeles, California, United States; 2 USC Ginsburg Institute for Biomedical Therapeutics, University of Southern California, Los Angeles, California, United States; 3 Roski Eye Institute, Keck School of Medicine, University of Southern California, Los Angeles, California, United States; 4 Center for Stem Cell Biology and Engineering, Neuroscience Research Institute, University of California, Santa Barbara, Santa Barbara, California, United States; 5 Department of Molecular Cellular and Developmental Biology, University of California, Santa Barbara, Santa Barbara, California, United States; 6 College of Creative Studies, Biology, University of California, Santa Barbara, Santa Barbara, California, United States; 7 Biomolecular Science and Engineering, University of California, Santa Barbara, Santa Barbara, California, United States; 8 Children's Hospital Los Angeles Vision Center, Los Angeles, California, United States; 9 Department of Physiology and Neuroscience, Keck School of Medicine, University of Southern California, Los Angeles, California, United States

**Keywords:** extracellular vesicle, stem cell–derived EV, EV isolation and characterization

## Abstract

**Purpose:**

Isolating extracellular vesicles (EVs) with high yield, replicable purity, and characterization remains a bottleneck in the development of EV therapeutics. To address these challenges, the current study aims to establish the necessary framework for preclinical and clinical studies in the development of stem cell–derived intraocular EV therapeutics.

**Methods:**

Small EVs (sEVs) were separated from the conditioned cell culture medium (CCM) of the human embryogenic stem cell–derived fully polarized retinal pigment epithelium (hESC-RPE-sEV) by a commercially available microfluidic tangential flow filtration (TFF) device ExoDisc (ED) or differential ultracentrifugation (dUC). The scaling and concentration capabilities and purity of recovered sEVs were assessed. Size, number, and surface markers of sEVs were determined by orthogonal approaches using multiple devices.

**Results:**

ED yielded higher numbers of sEVs, ranging from three to eight times higher depending on the measurement device, compared to dUC using the same 5 mL of CCM input. Within the same setting, the purity of ED-recovered hESC-RPE-sEVs was higher than that for dUC-recovered sEVs. ED yielded a higher concentration of particles, which is strongly correlated with the input volume, up to 10 mL (*r* = 0.98, *P* = 0.016). Meanwhile, comprehensive characterization profiles of EV surface markers between ED- and dUC-recovered hESC-RPE-sEVs were compatible.

**Conclusions:**

Our study supports TFF as a valuable strategy for separating sEVs for the development of intraocular EV therapeutics. However, there is a growing need for diverse devices to optimize TFF for use in EV preparation. Using orthogonal approaches in EV characterization remains ideal for reliably characterizing heterogeneous EV.

Extracellular vesicles (EVs) are membrane particles with a lipid bilayer released by almost every type of cell, constituting a distinct fraction of cell secretome.^1^^–^^5^ EVs transport a diverse array of biological cargo, not only reflecting the state of their parent cells but also playing an important role in modulating the functions of neighboring cells through intercellular communication. Therefore, EVs hold substantial therapeutic potential as a novel therapeutic platform to reshape molecular environments and leverage active components within the cell secretome.

While the development of EV therapeutics for eye diseases is in relatively early stages, ongoing studies involving stem cell–based cell replacement trials in humans are providing encouraging results, highlighting the significant therapeutic potential of stem cell–based EV therapy.^6^^–^^15^ For instance, the retinal pigment epithelium (RPE) plays crucial and specialized roles within the retina but is vulnerable to retinal diseases and lacks natural regenerative capabilities. Among the various strategies under investigation to address RPE cell dysfunction as an approach for treating AMD, the RPE secretome holds great potential, benefiting from technological advancements in differentiating RPE from pluripotent stem cells. These fully differentiated and polarized RPE cells have proven intraocular safety profiles from ongoing human clinical trials.^11^^–^^14^ The strength of the RPE secretome lies in employing a multimolecular approach, better supporting the multifunctional nature of the RPE, whereas an approach targeting a single molecule is often suboptimal in therapeutic efficacy. Therefore, there has been a rapidly increasing interest in the development of various stem cell–derived intraocular EV therapies. Furthermore, recent studies have demonstrated the broad therapeutic capabilities of EVs, including innate biocompatibility, high physical–chemical stability, and cell-selective fusion.^16^^–^^18^ Of the various EV classes, there has been a focused interest in the small EV (sEV, diameter ≈30–200 nm), which is often called the exosome, although sEV includes both exosomes that are released via the multivesicular body (MVB) and ectosomes that are generated from the cell surface.^7^^–^^10^^,^^19^

A few GMP-compliant sEV separation methods, such as serial filtration, tangential flow filtration (TFF) with or without gradient sedimentation, and size-exclusion chromatography (SEC), have been explored.^20^^–^^22^ However, due to the structural and molecular heterogeneity of naturally cell-secreted EVs, the development of a reproducible and scalable framework for EV isolation and characterization essential for translational applications has not been well established. While the yield and purity are two essential aspects to consider for sEV separation, the development of intraocular EV therapeutics demands a high concentration of particles due to the limited volume allowed for a single intraocular injection to avoid inadvertent intraocular pressure spikes above the intraocular perfusion pressure. These methods should also be feasible and replicable for scaling up for human applications. After recovering sEV, a comprehensive and reproducible sEV characterization strategy is also essential. This strategy is critical not only for biophysical identification of the recovered EVs but also for consistently capturing the unique molecular characteristics of each sEV group. Establishing these frameworks is equally important for identifying active components of sEV in preclinical studies, enabling the replication and prediction of study outcomes in a manner that is relevant to human applications.

In this study, we assessed ExoDiscovery (LabSpinner, Ulsan, South Korea), an automated centrifugal microfluidic system. It separates sEV from biofluids using a disposable TFF cartridge, ExoDisc (LabSpinner), equipped with nano-sized pores.^23^ We compared it to the differential ultracentrifugation (dUC) method to evaluate its scalability potential for preclinical and clinical studies in the development of intraocular sEV therapy. sEV preparation was generated from polarized RPE cells derived from human embryonic stem cells (hESC-RPEs), known for their established safety profile in humans.^14^^,^^15^ We examined both the yield and purity of sEV, demonstrating their potential for scaling up. Furthermore, we characterized the distinct surface protein profiles of sEV secreted by hESC-RPEs using comprehensive orthogonal approaches.

## Materials and Methods

### Cell Differentiation, Culture, and Characterization

Human embryonic stem cells (WA09; WiCell Research Institute, Madison, WI, USA) were spontaneously differentiated into RPE cells as previously described.^24^ Briefly, colonies of hESCs were manually passaged and seeded onto tissue culture plates coated with Matrigel hESC-Qualified Matrix (#354277; Corning, Corning, NY, USA), and they received biweekly medium exchanges of XVIVO-10 culture medium (#(BE) BP04-743Q; Lonza, Walkersville, MD, USA) for approximately 3 months. Pigmented regions were then manually isolated, expanded for two passages, and cryopreserved at 2 to 5 days postseed as a cellular suspension using CryoStor10 cryopreservation medium (#210102; BioLife Solutions, Bothell, WA, USA). Thawed cells were expanded using 10 µM Y-27632 (#1254; Tocris, Bristol, UK) until the eighth passage as previously described.^25^

The HEK293T (2 × 10^6^) cells were cultured in 10 mL high glucose Dulbecco's modified Eagle's medium (Thermo Fisher Scientific, Waltham, MA, USA) containing sodium pyruvate (110 mg/L), L-glutamine (200 mm), 10% fetal bovine serum, and 1% penicillin (10,000 U/mL)/streptomycin (10,000 µg/mL) in a 10-cm culture dish at 37°C in a 5% CO_2_ atmosphere. The passage number for HEK293T cells was between 7 and 10 in this study.

### Immunocytochemistry and Confocal Fluorescence Microscopy

hESC-RPEs, for immunocytochemistry, were cultured on sterile, Matrigel-coated, #1.5 coverslips (GG121.5PRE; Neuvitro, Vancouver, WA, USA) prior to fixation with 4% methanol-free formaldehyde in PBS for 20 minutes. Cells were subsequently permeabilized with 0.1% Triton X-100 for 10 minutes, blocked with 5% goat serum (Jackson ImmunoResearch, West Grove, PA, USA) and 1% BSA (Thermo Fisher Scientific) in PBS for 30 minutes, and incubated in the following primary antibodies overnight at 4°C: rabbit–anti-ZO1 (40–2200; Thermo Fisher Scientific, 1:200) and mouse–anti-RPE65 (MAB5428; Millipore-Sigma, Burlington, MA, USA, 1:200). Coverslips were washed three times with PBS followed by secondary antibody incubation in blocking buffer for 1 hour at room temperature. Secondary antibodies were Alexa Fluor 594 AffiniPure goat anti-rabbit IgG (111585144; Jackson ImmunoResearch, 1:200) and Alexa Fluor 488 AffiniPure goat anti-mouse IgG (115545062; Jackson ImmunoResearch, 1:200). Nuclei were stained for 10 minutes with Hoechst 33342 in PBS (2 µg/mL), washed three times with PBS, mounted in ProLong Gold antifade, and imaged on an Olympus FV1000 Spectral Confocal with a PLAPON-SC 60X oil objective (NA: 1.40) and excitation laser lines at 405, 488, 559, and 635 nm. Scale bars for fluorescence confocal images are 50 µm.

### Preparation of Conditioned Cell Culture Medium

To generate conditioned cell culture medium (CCM) from hESC-RPE evaluation, RPE cells were enzymatically passaged using TrypLE Select (#12563011; Gibco, Grand Island, NY, USA) per the manufacturer's instructions and seeded onto tissue culture plates coated with Matrigel hESC-Qualified Matrix (#354277; Corning) at a density of 70,000 cells/cm^2^. Viability of the cells at the time of plating was determined by trypan blue exclusion, and cultures exceeding 95% viability were used for the study. At 1 day postseed, cells were rinsed with DPBS (#14040141; Gibco) and fed with 4 mL XVIVO-10 culture medium (#(BE)BP04-743Q; Lonza) per well within a 6-well tissue culture plate. Medium was exchanged twice per week and supplemented with 10 µM Y-27632 (#1254; Tocris) for 10 to 15 days until the RPE cells attained cuboidal morphology. Starting at 11 days postseed and continuing twice per week until 22 days postseed, CCM was collected from four wells of a 6-well plate using a serological pipet, combined, and immediately frozen in a 50-mL Falcon centrifuge tube at −80°C until the downstream evaluation of sEV. Four replicate plates were used at each collection time point. The incubation period in which cells conditioned the medium ranged from 3 to 4 days at 37°C, 5% CO_2_. To generate CCM from HEK293T cells with 90% confluency (7 × 10^6^ cells), the medium was changed to 10 mL X-VIVO 10 serum-free media (Lonza) containing 1% penicillin (10,000 U/mL)/streptomycin (10,000 µg/mL) and maintained for another 4 days at 37°C in a 5% CO_2_ atmosphere to collect the conditioned medium. sEVs recovered from HEK293T cells using dUC were used as a nonocular tissue control in this study.

### Extracellular Vesicle Recovery

sEVs were separated by either ExoDisc (LabSpinner), a disposable TFF cartridge equipped with nanosized pores,13 mm in diameter with a pore size of 20 nm, or dUC.

sEV separation with ExoDisc (LabSpinner) was done following the manufacturer's protocol.^23^ In brief, different input volumes of CCM (1 mL, 3 mL, 5 mL, and 10 mL) were processed on an ExoDisc using the bench‐top operating machine (OPR‐1000; LabSpinner). Purified sEVs were collected from the collection chamber using 100 µL PBS and stored at −80°C until further use within 2 to 4 weeks. EV separation using dUC was completed as previously shown.^10^^,^^24^ Varying input volumes of CCM (5 mL, 15 mL, 20 mL, and 30 mL) were centrifuged at 25,000 × rpm (64,000 × *g*) for 60 minutes at 4°C in a Beckman Coulter Optima LE-80K (Brea, CA, USA) ultracentrifuge with rotor SW55 Ti. Then the supernatant was subjected to ultracentrifugation at 41,000 × rpm (173,000 × *g*) for 120 minutes at 4°C.

The samples used for the comparison between dUC and ExoDisc (ED) recovery were from the same CCM batches. All comparisons are based on triplicates, and each replicate (same for UC and ED) is from a different batch, within a specific age range. The final pellet (sEVs) was resuspended in 100 µL PBS and stored at −80°C until further use within 2 to 4 weeks.

### Biophysical Particle Analysis

The particle concentration and size distribution of recovered sEVs were measured by three different single-particle analysis platforms: NanoSight (Malvern Panalytical, Malvern, UK), ZetaView (Particle Metrix, Inning am Ammersee, Germany), and Flow Nanoanalyzer (NanoFCM, Xiamen, China) following the manufacturers’ protocols. In brief, for NanoSight (NS) analysis, the samples were diluted to obtain the optimal detection concentration of 10^8^ particles/mL; an automated syringe pump was used to achieve a constant flow speed of 25, and five videos were captured using camera level 14. The data were analyzed using NTA software 4.3 with detection threshold 7 and adjusted by the dilution factor. For ZetaView (NV) analysis, samples were diluted to achieve an ideal particle per frame value of 150 to 200 particles/frame. For each measurement, three cycles were performed by scanning 11 cell positions each and capturing 60 frames per position under the following settings: focus, auto; camera sensitivity for all samples, 85.0; shutter, 70; and cell temperature, 25°C. Following the data capture, the videos were subjected to analysis using the built-in ZetaView Software version 8.02.31 (Inning am Ammersee, Germany). For Flow Nanoanalyzer (FN) analysis, recovered sEVs were diluted in a 1:100 solution of PBS according to the manufacturer's protocol.^25^ The concentration of the samples was determined by calibrating the sample flow rate with a NanoFCM Quality Control Nanospheres solution (NanoFCM, Nottingham, UK), and the size distribution of the samples was calculated by referencing standard curves generated by a Silica Nanospheres Cocktail solution with diameters of 68, 91, 113, and 151 nm, respectively (NanoFCM). To increase the accuracy of readings, PBS was also analyzed as a background signal and subtracted from any following measurements. The collected data were then analyzed in the NanoFCM Profession V1.0 software to calculate and create the size and distribution histograms of the samples.

### Transmission Electron Microscopy

The size and morphology of recovered sEVs were visualized by negative-stained transmission electron microscopy (TEM) using a JEOL JEM-2100 (Tokyo, Japan) microscope mounted on a Gatan (Pleasanton, CA, USA) OneView IS camera. To prepare the thin formvar/carbon-coated 400-mesh copper EM grids (01754-F; Ted Pella, Redding, CA, USA) for imaging, 6 µL diluted sEV solution was loaded onto the grid and incubated at room temperature for 4 minutes. The sEV dilution was determined by the concentration of the sample. After incubation, the excess sEV solution was wicked using chromatography paper, and the grid was stained with 10 µL filtered 4% uranyl acetate solution for 3 minutes. After the staining, the excess 4% uranyl acetate solution was removed by contacting the grid edge with filter paper and allowed to dry for 10 minutes before storing the grid in an EM grid box for future observation by TEM at 80 kV.

### Protein Concentration Analysis

The recovered sEVs were lysed by mixing 5 µL of each sample with 5 µL of 2× RIPA buffer. Protein concentrations of the lysed sEV preparations were quantified using the Pierce BCA Protein Assay Kit (Thermo Fisher Scientific), following the manufacturer's protocols.

### MicroRNA Concentration Analysis

The RNA extraction from EVs was performed using an exoRNeasy midi kit (QIAGEN, Hilden, Germany) according to the manufacturer's protocol. In brief, preisolated EVs from samples using ED or UC (100 µL) were mixed with equal volume of a binding buffer (XBP) and added to the membrane affinity column. After discarding the flow-through using a centrifuge, a washing buffer (XWP) was added to the column, and the EVs were mixed with a lysis reagent (QIAzol) passing through the membrane and extracted to the bottom of the tube. Then, chloroform was added to remove the phenol component, and the clear layer containing the isolated RNA was separated through centrifugation. After several washing steps, total RNA from samples were finally obtained. The purity and quantity of the isolated total RNA were measured using a Nanodrop 2000 spectrophotometer (Thermo Fisher Scientific). Quantification of total microRNAs (miRs) was performed using the Qubit 4 Fluorometer and Qubit microRNA Assay Kit (Invitrogen, Thermo Fisher Scientific) according to the manufacturer's instructions.

### Single-Particle Interferometric Reflectance Imaging Sensing: ExoView Analysis

The analysis was conducted using single-particle interferometric reflectance imaging sensing (SP-IRIS) with the ExoView R100 system and the ExoView Human Tetraspanin Kit (NanoView Biosciences, Brighton, MA, USA). Each sample was incubated on an ExoView Tetraspanin Chip for 16 hours at room temperature, followed by three washes in solution A (ExoView Human Tetraspanin Kit; NanoView Biosciences). Prediluted immunocapture antibodies (anti-CD9 CF488, anti-CD81 CF555, and anti-CD63 CF647) were used at a 1:500 dilution in solution A. To achieve the correct antibody concentration, 250 µL of the antibody solution was mixed with the remaining 250 µL of solution A after chip washing, resulting in a final antibody dilution of 1:1000 for incubation. After a 1-hour incubation at room temperature, the chips were washed, dried, and imaged using the ExoView R100 reader and ExoView Scanner 3.0 acquisition software, and the data were subsequently analyzed using the ExoView Analyzer 3.0.

### Bead-Based Multiplex Flow Cytometry Assay (MACSPlex) Analysis

To assess the expanded surface protein markers of sEVs, a MACSPlex human Exosome kit (Miltenyi Biotec, Bergisch-Gladbach, Germany) was used following the manufacturer's protocol. The sEVs were captured using 37 distinct surface marker antibodies (CD1c, CD2, CD3, CD4, CD8, CD9, CD11c, CD14, CD19, CD20, CD24, CD25, CD29, CD31, CD40, CD41b, CD42a, CD44, CD45, CD49e, CD56, CD62p, CD63, CD69, CD81, CD86, CD105, CD133.1, CD142, CD146, CD209, CD326, HLA-ABC, HLA-DR DP DQ, MCSP, ROR1, and SSEA-4) simultaneously and include the two isotype controls (mIgG1 and REA control) corresponding to the antibodies conjugated with fluorescent beads and then analyzed via flow cytometry. The samples were run on a Cytek Aurora Flow Cytometer (Cytek Biosciences, Fremont, CA, USA) and analyzed with SpectroFlo software (Cytek Biosciences).

### Statistical Analysis

Unless specified otherwise, data are presented as mean ± SD. Statistical analysis and graph plotting were performed using GraphPad Prism (GraphPad Software, La Jolla, CA, USA). Student's *t*-test was utilized to compare the two groups, and significance was defined as *P* < 0.05.

## Results

### sEV Isolation and Biophysical Characterization Compared in Multiple Devices

**Figure 1.**
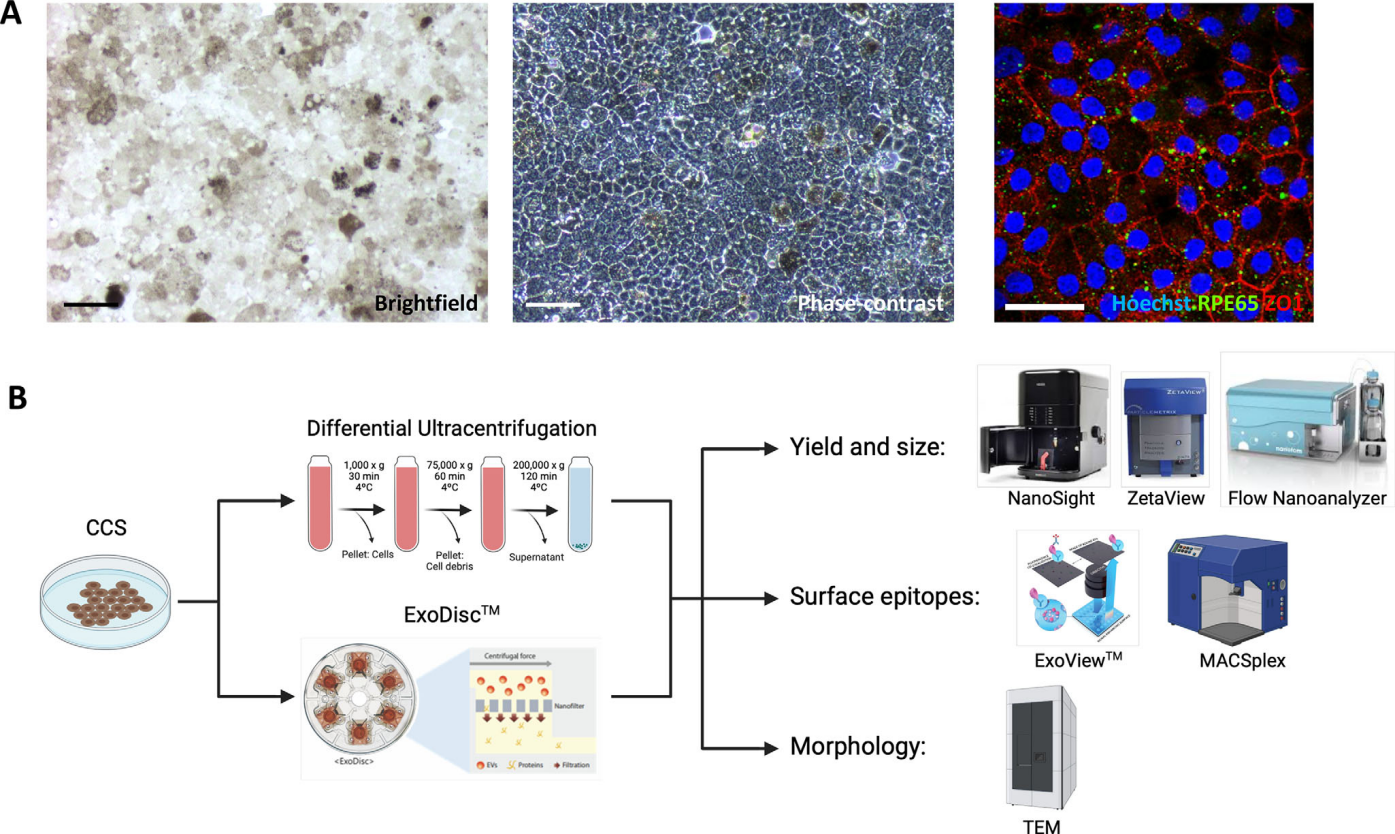
(**A**) Representative images of pigmented human embryogenic stem cell–derived fully differentiated and polarized RPE cells (hESC-RPEs) exhibiting a cobblestone morphology. The *left image* is captured using brightfield microscopy, while the *middle image* employs phase contrast microscopy. Further characterization of RPE cells was conducted by staining the cells with Hoechst (*blue*), RPE65 (*green*), and ZO1 (*red*) (*right image*). (**B**) A schematic depiction of the experimental design, illustrating the overall research approach and methodology. *Scale bar*: 100 µm.

**Figure 2.**
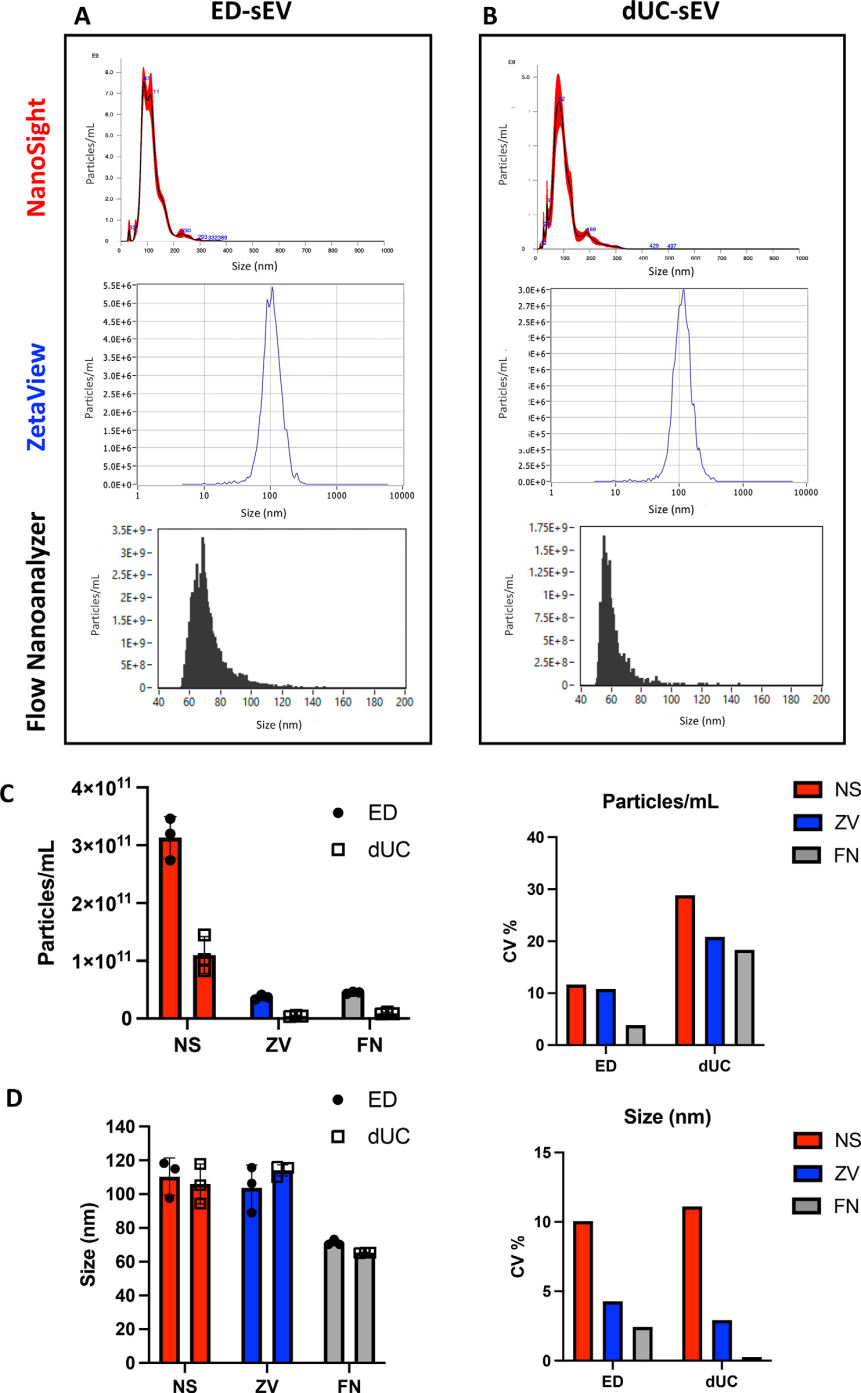
Comparison of sEV size and concentration analysis using different NTA methods. (**A**) Representative size versus concentration distribution graphs of sEVs isolated by ED as analyzed by NS (*top*), ZV (*middle*), and FN (*bottom*). (**B**) Representative size versus concentration distribution graphs of sEVs isolated by dUC as analyzed by NS (*top*), ZV (*middle*), and FN (*bottom*). (**C**) When comparing sEV particle concentration, NS demonstrates a yield of sEVs 8.5 times higher than ZV and 7 times higher than the FN. (**D**) Analyzing sEV particle size reveals that the FN measures a mean particle size 1.5 times smaller than those measured by NS and ZV. Results were obtained from a starting volume of 5 mL for both ED and dUC recovery methods.

sEVs were recovered from CCM of fully characterized hESC-RPEs (Figs. 1A, 1B). For ED-recovered sEV, the quantified particle numbers were 3.13 × 10^11^, 3.7 × 10^10^, and 4.5 × 10^10^ particles/mL measured by NanoSight (NS), ZetaView (ZV), and Flow Nanoanalyzer (FN), respectively (^1^Figs. 2A, 2C). For UC-recovered sEVs, the quantified particle numbers were 1.1 × 10^11^, 4.47 × 10^9^, and 9.17 × 10^9^ particles/mL measured by NS, ZV, and FN respectively (Figs. 2B, 2C). Despite variations in particle number measurements among the three devices, ED consistently yielded higher numbers of sEVs, ranging from three to eight times higher, compared to dUC using the same 5 mL of CCM input. For ED-recovered sEVs, the mean diameter (size) of particles was 110.27, 110.67, and 71.2 nm measured by NS, ZV, and FN, respectively (Fig. 2D). The coefficient of variation (CV) of particle number measurements in ExoDisc (ED) recovery was 11.6%, 10.44%, and 3.86% for NS, ZV, and FN, respectively. In dUC recovery, it was 28.83%, 20.8%, and 18.32% for NS, ZV, and FN, respectively (Fig. 2C). Regarding particle size, the CV measured 10.05%, 4.28%, and 2.43% for NS, ZV, and FN, respectively, during ED recovery. For dUC recovery, the CV of particle size was 11.11%, 2.92%, and 0.27% for NS, ZV, and FN, respectively (Fig. 2D).

### Transmission Electron Microscopy

**Figure 3.**
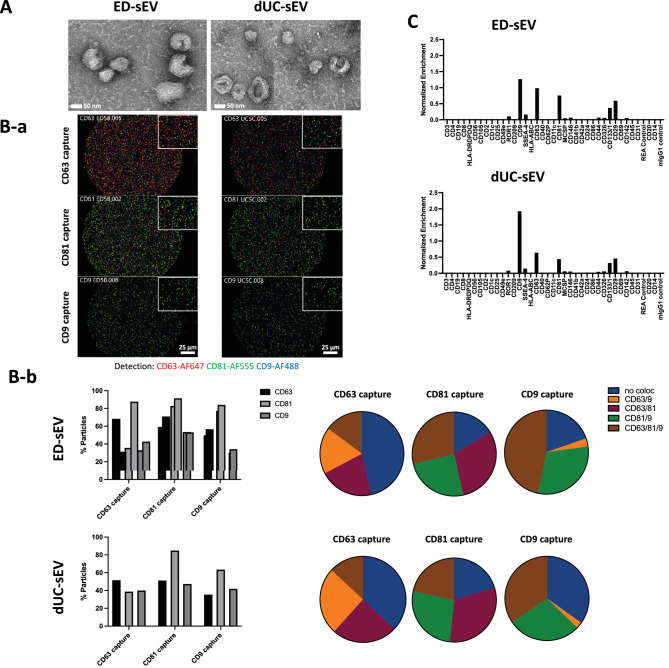
Comparative analysis of sEV characteristics using different isolation methods. (**A**) TEM images of ED-derived and dUC-derived sEVs, providing a visual comparison. (**B**) Colocalization analysis of tetraspanin (CD81, CD9, and CD63) subgroups within sEVs recovered using or ED or dUC. (**B**-**a**) Shown are representative fluorescent images detected using fluorescent-conjugated antibodies. (**B**-**b**) The distribution of tetraspanin subpopulations in ED-sEVs and dUC-sEVs. (**C**) Results from the MACSPlex assay conducted on ED-sEVs and dUC-sEVs. **P < 0.01, ****P < 0.0001.

In the TEM images of hESC-RPE-sEVs, most of the particles showed a cup-shaped morphology with varying sizes, ranging from 30 to 120 nm, similar in both ED- versus dUC-recovered sEVs (Fig. 3A). HEK293T cell-derived sEVs displayed a similar cup-shaped morphology size range (Supplementary Fig. S1B).

### Characterization of Tetraspanin Expression

The tetraspanin subpopulation profiles in both ED- and dUC-recovered hESC-RPE-sEVs were similar (Fig. 3B). This preserved tetraspanin subpopulation profile in hESC-RPE-sEVs, regardless of the recovery method (ED versus dUC), was also comparable to that in HEK293T-sEVs (Supplementary Fig. S1D).

### EV Surface Epitopes

Of the 37 EV surface proteins tested using the MACSPlex assay, 12 proteins, including ROR1, CD9, SSEA-4, CD63, CD81, MCSP, CD146, CD44, CD326, CD133/1, CD29, and CD142, were found to be expressed in the hESC-RPE-sEVs (Fig. 3C). Of these 12 expressed proteins, CD133/1 and CD29 were found to be highly expressed in addition to all three major tetraspanins, including CD9, CD81, and CD63. In contrast, this expression pattern was distinctly different from that of HEK293T cell-derived sEVs (Supplementary Fig. S1E).

### Scaling and Concentration Capabilities

**Figure 4.**
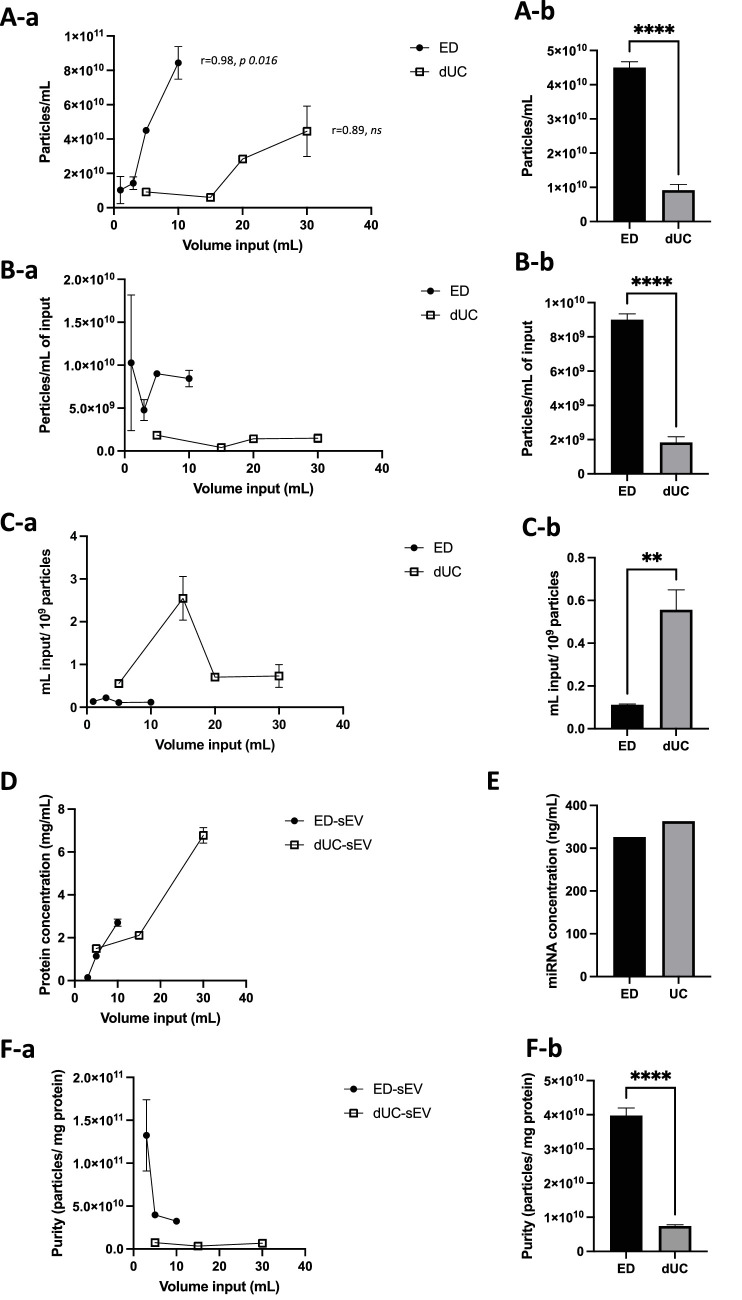
Comparison of ED and dUC for sEV isolation based on FN. (**A-a**, **A-b**) ED offers a more efficient scaling potential, requiring a lower initial sample input volume to yield higher sEV quantities in comparison to dUC. *Left*: the graph shows the comparative input volume required for sEV isolation. *Right*: sEV concentration from a 5-mL initial sample input volume was 390.6% higher with ED than with dUC. (**B-a**, **B-b**) ED allows for the recovery of a larger number of sEVs per unit initial sample input volume compared to dUC. *Left*: the graph illustrates the sEV recovery efficiency. ED can recover approximately four times as many sEV per 1 mL of initial sample input volume as dUC. *Right*: results were obtained from an initial volume of 5 mL. (**C-a**, **C-b**) ED requires a smaller sample volume for the recovery of 10^9^ sEV particles when compared to dUC. *Left*: the graph displays the volume required for retrieving 10^9^ particles. dUC necessitates 400.3% more mL of input volume to recover 10^9^ particles compared to ED. *Right*: results were obtained from a starting volume of 5 mL. (**D**) The protein concentration of ED-sEVs and dUC-sEVs was evaluated across three different initial sample input volumes (5 mL, 15 mL, and 30 mL and 3 mL, 5 mL, and 10 mL for dUC and ED, respectively). (**E**) The miR concentration from 5-mL input volume was not different between ED- and dUC-sEVs. (**F-a**) Assessing sample purity through the particle/protein ratio, with various initial sample input volumes. (**F-b**) Specifically, 5-mL input volume revealed a 5.4-fold higher purity ratio for ED compared to dUC. **P < 0.01, ****P < 0.0001.

ED required as little as 1 mL of CCM volume to recover 1 × 10^9^ particles/mL of sEVs. Increasing the input volume of CCM resulted in a higher concentration of sEVs with a correlation coefficient of *r* = 0.98 (*P* = 0.016), while the correlation analysis for the dUC method did not show statistical significance suggestive of limited capacity for the concentration of sEVs. ED required a much smaller initial sample input volume and achieved a 390.6% increase in sEV concentration from a 5-mL initial sample input volume compared to dUC (*P* < 0.0001) (Fig. 4A). ED recovered about five times as many sEVs per 1 mL of initial sample input volume compared to dUC (*P* < 0.001) (Fig. 4B). Notably, ED significantly reduced the needed sample volume to retrieve 1 × 10^9^ sEV particles compared to dUC, where dUC demanded a 400.3% larger input volume (*P* = 0.0012).

### Purity of sEV Samples Based on Protein, miR, and Particle/Protein Ratio

The total protein concentration of sEV preparation recovered from different initial input volumes (3 mL, 5 mL, and 10 mL for ED and 5 mL, 15 mL, and 30 mL for dUC) was analyzed (Fig. 4D). Specifically, ED- and dUC-recovered sEVs from 5 mL of CCM demonstrated protein concentrations of 1.14 mg/mL and 1.49 mg/mL, respectively (*P* < 0.0001). Meanwhile, the total miR concentration of ED- and dUC-recovered sEVs from 5 mL of CCM were comparable (0.326 vs. 0.353 ng/µL, respectively) (Fig. 4E). The calculated particle-to-protein ratio to determine the purity of recovered sEV preparations was 3.98 × 10^10^ particles/mg protein and 7.43 × 10^9^ particles/mg protein in ED- and dUC-recovered sEV, respectively (*P* < 0.0001), suggesting that the purity of ED-recovered hESC-RPE-sEVs is higher than dUC-recovered hESC-RPE-sEVs (Fig. 4F-b). The results were confirmed through a repetition of the analysis using NanoSight and ZetaView devices, providing further assurance of the reliability and consistency of the findings (Supplementary Figs. S2D and S3D).

## Discussion

Upfront methodologic challenges in developing stem cell–derived EV therapeutics are multidimensional yet interrelated. Different cell lines naturally secrete diverse particles to their CCM. Thus, it is a necessary step to establish reliable methodologies for separating and characterizing EVs by size, numbers, morphology, total protein, and EV surface markers to identify both biophysical and molecular properties. However, multiple previous studies demonstrated that existing methodologies vary between methods or devices and often provide qualitative rather than quantitative results.^26^^–^^31^ Beyond these common challenges in EV research, the development of intraocular stem cell–derived EV therapeutics specifically requires a separation method that is not only feasible and replicable but also capable of scaling up to recover particles, maintaining high concentration, yield, and purity.

In this study, we investigated the yield and purity of sEVs recovered from hESC-RPE CCM by ED, a disposable TFF cartridge, and dUC. To evaluate the scalability potential with a high concentration of sEVs, we examined the sEV outputs per various input volumes of CCM. Our results showed that ED can yield a higher concentration of particles with 5-mL input volumes of CCM while also maintaining higher purity (particles/mg protein) as compared to dUC and comparable total miR concentration. A previous study on ED reported a high yield; however, when extracting sEVs from 30-mL input volumes of CCM, the study showed only moderate purity due to a high presence of soluble proteins. On the other hand, sEVs isolated from urine exhibited high purity, while those from plasma had lower purity.^32^ The authors speculated that an abundance of small particles in higher-volume input might overwhelm the filter, forming a "bed" and leading to clogging.^33^ Based on our results, we speculate that multiple variables could affect the protein fractions of CCM. We used a smaller volume (5 mL) of CCM input compared to 30 mL. Additionally, we employed X-VIVO 10 instead of a regular medium containing extracellular vesicle–free fetal bovine serum (EV-free FBS). X-VIVO 10 is a serum-free medium suitable for fully differentiated and polarized RPE. This choice may contribute to a lower fraction of soluble proteins, potentially reducing the risk of clogging the microfilter. Furthermore, CCM from various cell lines (e.g., tumor cell versus stem cell), containing different protein compositions, may affect the recovery performance of ED. To evaluate the scaling potential of ED, we examined the sEV outputs and purity for various input volumes of CCM. Our results show that ED can yield a higher concentration of particles, which is strongly correlated with the input volume, up to 10 mL (Fig. 4A, *r* = 0.98, *P* = 0.016). Additionally, it maintains higher purity (particles/mg protein) compared to dUC. However, the purity tends to decrease with increased input volumes of CCM (Fig. 4A). In addition to its favorable scalability potential, ExoDisc utilizes an automated small tabletop centrifuge that makes EV recovery highly time-efficient (30 minutes with ED vs. 450 minutes with dUC). It offers additional advantages through its available sterilized, single-use disc with a filtering membrane, making it a promising method for preclinical and clinical studies. This particular device may be better suited for generating small volumes with the high concentration required for intraocular EV therapeutics, given its handling limit for small volumes. We observed that the filtering membrane started to clog at around 10 mL of input volume of CCM of hESC-RPE. Scaling up could be achieved by implementing a larger-sized microfiltration system. Meanwhile, the current version of the device lacks temperature control, and the EV preparation was conducted at room temperature, with the possibility of additional heat during the spinning of the rotor.

To characterize the retrieved sEVs from both ED and dUC, we assessed particle numbers and size using three orthogonal single-particle analysis platforms: NanoSight (Malvern Panalytical), ZetaView (Particle Metrix), and Flow Nanoanalyzer (NanoFCM). Across all three devices, ED consistently yielded significantly higher particle counts than dUC. However, the absolute numbers of measured particles recovered from ED varied, ranging from three to eight times higher than dUC, depending on the analysis platform. NS tended to report higher particle numbers as compared to ZV or FN, in which particle numbers were similar. While the sizes of particles recovered from ED and dUC were similar, NS and ZV reported larger particle sizes than FN. Regarding the measurement consistency within each device, FN had significantly less variation in both particle number and size as compared to NS and ZV. NS and ZV are nanoparticle tracking analysis (NTA) devices that utilize optical methods to track single particles, determine their sizes, and count them. On the other hand, FN utilizes nanoflow cytometry measurement, a flow-based technique that detects nanoparticles through simultaneous single-particle light scattering and fluorescence emission intensity detection. A previous study investigated EVs separated from CCM by dUC and demonstrated that NS measured significantly higher particle concentrations of EV preparations as compared to ZV.^34^ Another study showed that the EV count differences between NS and FN were about 10-fold.^35^ Although NS and ZV utilize the same principle of Brownian motion to visualize and calculate the concentration of nanoparticles in solution, each NTA device can provide different numbers and sizes of nanoparticles due to variations in hardware and software composition. In our study, TEM analysis of both ED- and dUC-recovered hESC-RPE-sEVs revealed heterogeneous particles with a typical cup-shaped structure, ranging from 30 to 120 nm in diameter. Although the size range of EVs measured by all three devices fell within the 30- to 120-nm range, the mean particle size and size distribution indicate that the FN measures a mean particle size 1.5 times smaller. In contrast, ZV and NS size measurements were more skewed toward the upper limit. The limit of detection or discrepancy across the devices can also be related to the EV preparation methods, as observed in our studies with ED and dUC. The CV of both number and size was lower in all three devices when measuring ED-recovered sEVs than dUC-recovered sEVs (Figs. 2C, 2D and Fig. 4D). Therefore, the cell or tissue source of EVs is also likely to influence the measurements of each device. While discussing the detailed biophysics of each device is beyond the scope of this study, we have established replicable particle counts and sizes of hESC-RPE-sEVs by utilizing an orthogonal approach to quantify the numbers and sizes of EVs.^36^

We further characterized a distinctive EV surface protein signature by employing two complementary methods: SP-IRIS for tetraspanin coexpression analysis at the single-vesicle resolution and bead-based multiplex flow cytometry assay (MACSPlex) for a comprehensive 37-plex surface marker profiling of EVs.

Therefore, these two analyses appear to overlap in tetraspanin assessment, but they are not directly comparable due to different imaging methodologies (single particle versus beads based).

In our study, the SP-IRIS analysis of hESC-RPE-sEVs revealed heterogeneity in the expression and coexpression of tetraspanins (CD81, CD9, and CD63). This signature was consistent between hESC-RPE-sEVs recovered using either ED or dUC and comparable to the tetraspanin subpopulation pattern of sEVs derived from HEK 293T, a nonocular reference cell. Although SP-IRIS provided valuable insights into tetraspanin expression at the single vesicle level, it had limitations in capturing a broad range of pan-EV biomarkers essential for robust characterization of each EV group's unique signature.^37^ In the MACSPlex analysis, we found additional markers like CD133/1, CD29, ROR1, SSEA-4, MCSP, CD146, CD44, CD236, and CD142, alongside the abundant CD81, CD9, and CD63. Among these, SSEA-4 (stage-specific embryonic antigen 4), MCSP (melanoma-associated chondroitin sulfate proteoglycan), and CD146 (melanoma cell adhesion molecule), despite their low expression, suggest the stem cell origin of hESC-RPE-EVs.^38^ The semiquantified unique expression of these markers in hESC-RPE-sEVs remained consistent between ED and dUC recovered sEVs. In contrast, EVs from HEK 293T cells displayed their own distinct epitope profiles.

In addition to previous reports on TFF, the uniqueness of our study lies in the proposed workflow framework necessary for preclinical and clinical studies in developing stem cell–derived intraocular therapeutics. This framework includes (1) testing the yield, purity, and scalability capacity of an EV recovery method that is potentially good laboratory practice (GLP) and cGMP compatible and (2) using reproducible orthogonal methods for EV biophysical and surface epitope characterization, which is necessary for precisely defining the specific characterization of EV products. Our study reaffirms TFF as a valuable strategy for developing EV therapeutics, especially given the high concentration demands of intraocular therapeutics. Consequently, there is an increasing need for diverse devices to optimize TFF for use in EV preparation.

Importantly, our study does not suggest the universal superiority of any specific device over others. It is evident that CCM from different cell lines under different culture conditions should be separately optimized before further studying their therapeutic effects. Additionally, the different sample sources such as ocular biofluids, including tear, aqueous humor, and vitreous humor, for diagnostic development, may require further optimization in the EV recovery and characterization strategies.

The present study has limitations. We used dUC without combining it with SEC as a reference method. While the combination of dUC and SEC offers better purification of EVs, the addition of SEC significantly reduces their yield, complicating the direct comparison between ExoDisc and dUC. Additionally, the term *purity* does not imply that the preparations contain only pure EVs; instead, it is a “purity index.” Furthermore, identifying the active components of sEVs in diverse therapeutic applications is a crucial aspect of advancing intraocular EV therapeutics, alongside establishing a framework for EV isolation and characterization.

While additional studies are needed to investigate the active component of the cell secretome and understand its mechanism of action—whether it is pure EV or an EV-enriched fraction—we believe our study provides valuable insights for developing workflows to create individualized intraocular EV therapeutic strategies.

## Supplementary Material

Supplement 1
