# Supplementary material for: Isolation and Characterization of Extracellular Vesicles Through Orthogonal Approaches for the Development of Intraocular EV Therapy
Source: Invest Ophthalmol Vis Sci. 2024 Mar 11;65(3):6. doi: 10.1167/iovs.65.3.6 (PMC10929743; doi:10.1167/iovs.65.3.6)
Supplement: Supplement 1 [file iovs-65-3-6_s001.pdf]

Supplementary Figure. 1. Detailed characterization of HEK293T-sEV recovered using differential ultracentrifugation. **(A)** Size versus concentration distribution of sEVs **(B)**Transmission electron microscopy (TEM). **(C)** Colocalization analysis of tetraspanin (CD81, CD9, and CD63) subgroups within sEVs. **(C-a)** Shown are representative fluorescent images detected using fluorescent-conjugated antibodies. **(C-b)** The distribution of tetraspanin subpopulations in sEV. **(D)** Results from the MACSPlex assay.

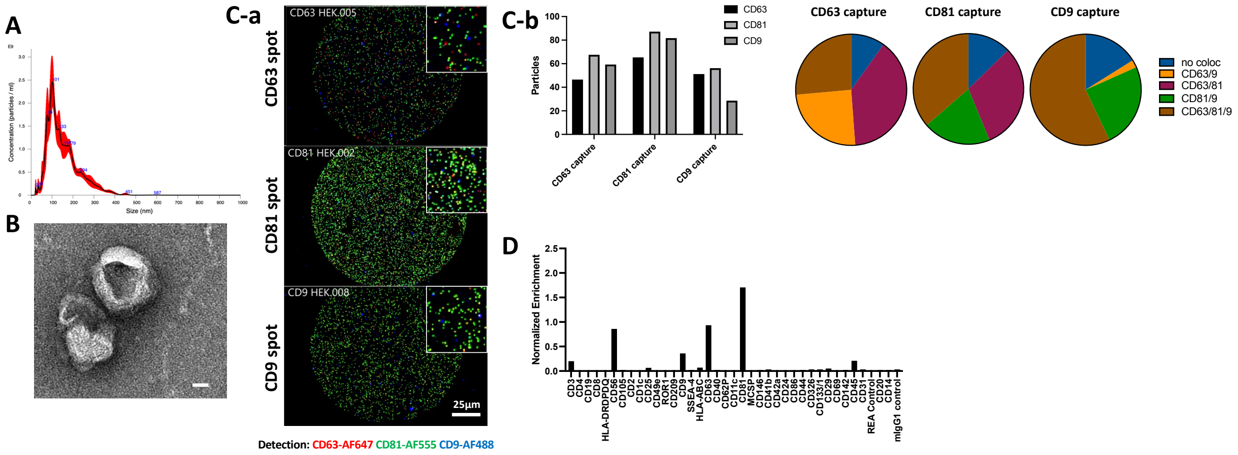

Supplementary Figure. 2. Comparison of ExoDisc® (ED) and Differential Ultracentrifugation (dUC) for sEV isolation based on NanoSight. (**A**) ED offers a more efficient scaling potential, demanding a lower initial sample input volume to yield higher sEVquantities in comparison to dUC. (**A-a**) The graph shows the comparative input volume required for sEV isolation. (**A-b**) sEV concentration from a 5 mL initial sample input volume was 184.8% higher with ED than with dUC. (**B**) ED allows for the recovery of a larger number of sEVs per unit initial sample input volume compared to dUC. (**B-a**) The graph illustrates the sEV recovery efficiency. (**B-b**) ED can recover approximately three times as many sEVs per 1 mL of initial sample input volume as dUC. Results were obtained from an initial volume of 5 mL. (**C**) ED demands a smaller sample volume for the recovery of 10^9 sEV particles when compared to dUC. (**C-a**) The graph displays the volume required for retrieving 10^9 particles. (**C-b**) dUC necessitates 197.3% more mL of input volume to recover 10^9 particles compared to ED. Results were obtained from a starting volume of 5 mL (right). (**D-a**) Assessing sample purity through the particle/protein ratio, with various initial sample input volumes. (**D-b**) Specifically, 5 mL input volume revealed a 2.8-fold higher purity ratio for ED compared to dUC. ** $p < 0.01$, **** $p < 0.0001$

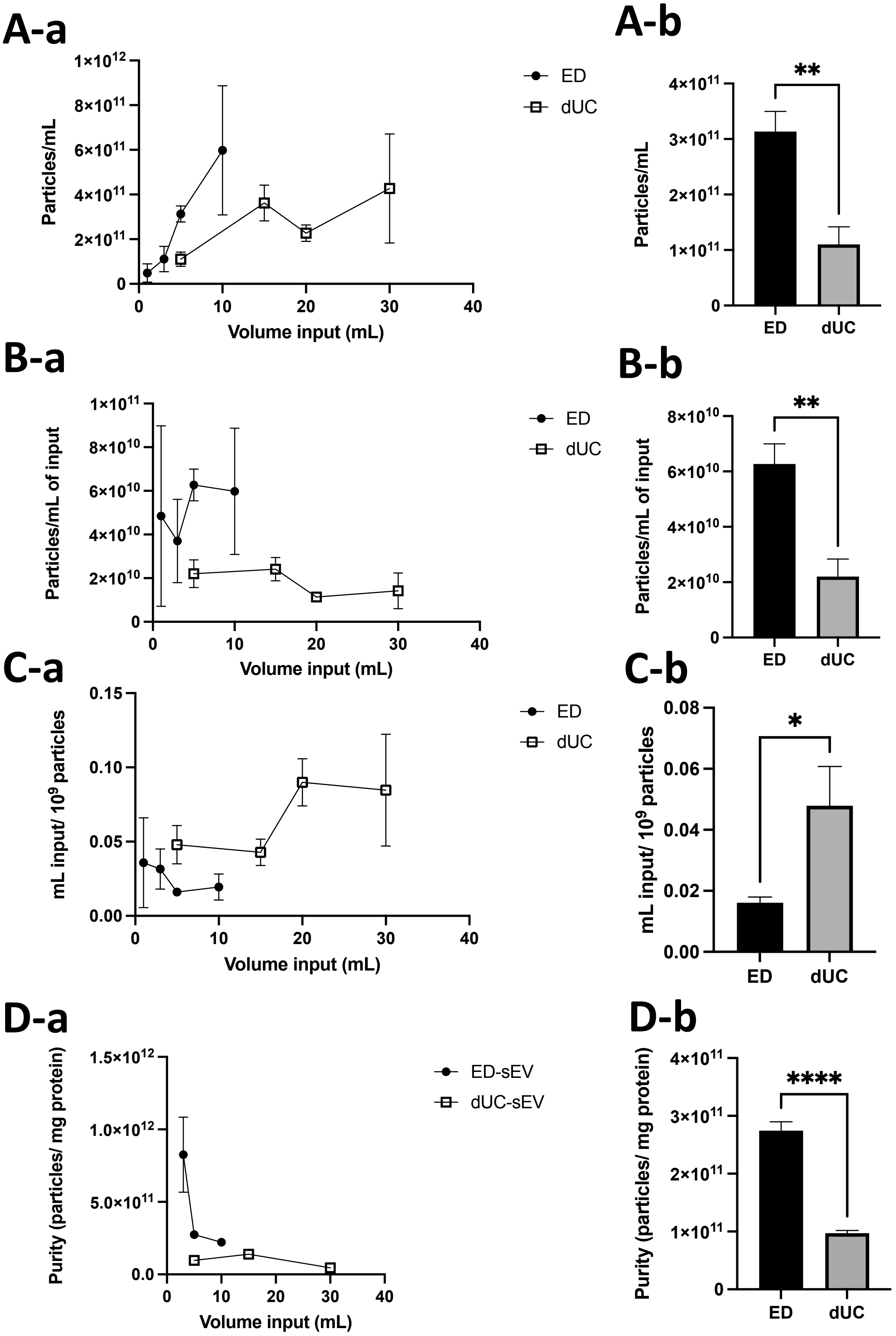

A-a
Particles/mL
Volume input (mL)
ED
dUC
A-b
**
B-a
Particles/mL of input
B-b
C-a
mL input/ 10^9 particles
C-b
*
D-a
Purity (particles/ mg protein)
ED-sEV
dUC-sEV
D-b
****

Supplementary Figure. 3. Comparison of ExoDisc® (ED) and Differential Ultracentrifugation (dUC) for sEV isolation based on ZetaView. (**A**) ED offers a more efficient scaling potential, demanding a lower initial sample input volume to yield higher sEVquantities in comparison to dUC. (**A-a**) The graph shows the comparative input volume required for sEV isolation. (**A-b**) sEV concentration from a 5 mL initial sample input volume was 728.4% higher with ED than with dUC. (**B**) ED allows for the recovery of a larger number of sEVs per unit initial sample input volume compared to dUC. (**B-a**) The graph illustrates the sEV recovery efficiency. (**B-b**) ED can recover approximately three times as many sEVs per 1 mL of initial sample input volume as dUC. Results were obtained from an initial volume of 5 mL. (**C**) ED demands a smaller sample volume for the recovery of 10^9 sEV particles when compared to dUC. (**C-a**) The graph displays the volume required for retrieving 10^9 particles. (**C-b**) UC necessitates 744.6% more mL of input volume to recover 10^9 particles compared to ED. Results were obtained from a starting volume of 5 mL. (**D-a**) Assessing sample purity through the particle/protein ratio, with various initial sample input volumes. (**D-b**) Specifically, 5 mL input volume revealed an 8.8-fold higher purity ratio for ED compared to dUC. ** $p < 0.01$, **** $p < 0.0001$

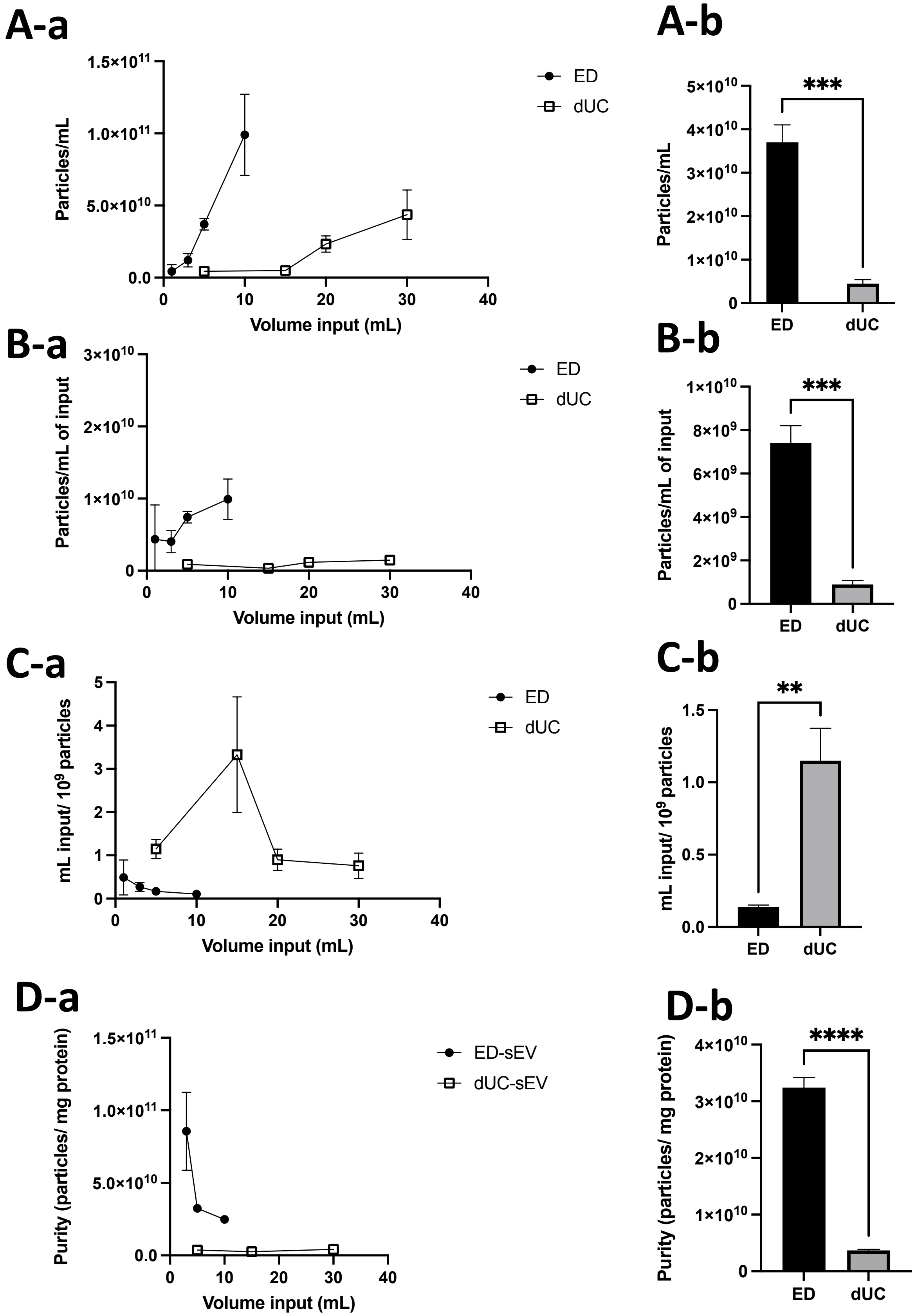
A-a
Particles/mL
1.5×10^11
1.0×10^11
5.0×10^10
0.0
0
10
20
30
40
Volume input (mL)
ED
dUC
A-b
***
Particles/mL
5×10^10
4×10^10
3×10^10
2×10^10
1×10^10
0
ED
dUC
B-a
Particles/mL of input
3×10^10
2×10^10
1×10^10
0
0
10
20
30
40
Volume input (mL)
ED
dUC
B-b
***
Particles/mL of input
1×10^10
8×10^9
6×10^9
4×10^9
2×10^9
0
ED
dUC
C-a
mL input/ 10^9 particles
5
4
3
2
1
0
0
10
20
30
40
Volume input (mL)
ED
dUC
C-b
**
mL input/ 10^9 particles
1.5
1.0
0.5
0.0
ED
dUC
D-a
Purity (particles/ mg protein)
1.5×10^11
1.0×10^11
5.0×10^10
0.0
0
10
20
30
40
Volume input (mL)
ED-sEV
dUC-sEV
D-b
****
Purity (particles/ mg protein)
4×10^10
3×10^10
2×10^10
1×10^10
0
ED
dUC
